# Methylation Sensitive Amplification Polymorphism Sequencing (MSAP-Seq)—A Method for High-Throughput Analysis of Differentially Methylated CCGG Sites in Plants with Large Genomes

**DOI:** 10.3389/fpls.2017.02056

**Published:** 2017-11-30

**Authors:** Karolina Chwialkowska, Urszula Korotko, Joanna Kosinska, Iwona Szarejko, Miroslaw Kwasniewski

**Affiliations:** ^1^Centre for Bioinformatics and Data Analysis, Medical University of Bialystok, Bialystok, Poland; ^2^Department of Genetics, University of Silesia in Katowice, Katowice, Poland; ^3^Department of Medical Genetics, Medical University of Warsaw, Warsaw, Poland

**Keywords:** DNA methylation, MSAP, methylome analysis, new technique, next generation sequencing, large genomes

## Abstract

Epigenetic mechanisms, including histone modifications and DNA methylation, mutually regulate chromatin structure, maintain genome integrity, and affect gene expression and transposon mobility. Variations in DNA methylation within plant populations, as well as methylation in response to internal and external factors, are of increasing interest, especially in the crop research field. Methylation Sensitive Amplification Polymorphism (MSAP) is one of the most commonly used methods for assessing DNA methylation changes in plants. This method involves gel-based visualization of PCR fragments from selectively amplified DNA that are cleaved using methylation-sensitive restriction enzymes. In this study, we developed and validated a new method based on the conventional MSAP approach called Methylation Sensitive Amplification Polymorphism Sequencing (MSAP-Seq). We improved the MSAP-based approach by replacing the conventional separation of amplicons on polyacrylamide gels with direct, high-throughput sequencing using Next Generation Sequencing (NGS) and automated data analysis. MSAP-Seq allows for global sequence-based identification of changes in DNA methylation. This technique was validated in *Hordeum vulgare*. However, MSAP-Seq can be straightforwardly implemented in different plant species, including crops with large, complex and highly repetitive genomes. The incorporation of high-throughput sequencing into MSAP-Seq enables parallel and direct analysis of DNA methylation in hundreds of thousands of sites across the genome. MSAP-Seq provides direct genomic localization of changes and enables quantitative evaluation. We have shown that the MSAP-Seq method specifically targets gene-containing regions and that a single analysis can cover three-quarters of all genes in large genomes. Moreover, MSAP-Seq's simplicity, cost effectiveness, and high-multiplexing capability make this method highly affordable. Therefore, MSAP-Seq can be used for DNA methylation analysis in crop plants with large and complex genomes.

## Introduction

DNA methylation is an epigenetic mechanism that influences gene expression, transposon mobility and genome integrity. Additionally, methylation affects the chromatin structure and controls its condensation in the nucleus (Richards and Elgin, 2002). Epigenetic modifications are generally defined as stable changes that do not involve DNA sequence alteration but result from the addition of specific chemical substituents to nucleotide bases in DNA or histone protein tails (Berger et al., 2009). DNA methylation is a modification resulting from the covalent addition of a methyl group to the fifth position of the aromatic ring in cytosine. The addition and removal of DNA methylation can be induced by both internal and external stimuli (Meyer, 2015).

In plants, DNA methylation occurs commonly within three sequence contexts: CG, CHG and CHH (where H is A, C, or T); however, it varies depending on the level and pattern found within different genomic and intergenic regions. The methylome of the model plant *Arabidopsis thaliana* has been extensively studied, although its DNA methylation levels and patterns are not shared by all plants. This phenomenon is observed because *A. thaliana* has a small genome size with low repetitive element content in addition to dissimilarities within methylating/demethylating enzymes (Kapazoglou et al., 2013; Yamauchi et al., 2014). One study observed that 24% of CG, 6.7% of CHG and 1.7% of CHH sequences were methylated in *A. thaliana* using bisulfite treatments coupled with global next generation sequencing (Cokus et al., 2008). Alternatively, in the *Oryza sativa* genome, which is three times larger, 59% of CG, 21% of CHG, and 2.2% of CHH sequences were methylated (Feng et al., 2010). Additionally, in the 20 times larger *Zea mays* genome, 86% of CG, 74% of CHG, and 5.4% of CHH sequences were methylated (Gent et al., 2013).

Many techniques have been developed to analyze DNA methylation and its alterations and can be classified as general or specific. General DNA methylation analyses, such as the popular HPLC or ELISA-based methods, determine the total level of DNA methylation within a genome, whereas specific methods identify regional changes within short sequences or even particular cytosines. Specific DNA methylation analyses are divided into three types: (1) methods based on antibodies or specific proteins that exhibit affinity to methylcytosine; (2) methods that apply methyl-sensitive restriction enzymes, and (3) methods involving the treatment of DNA with sodium bisulfite, which converts unmethylated cytosine to uracil (Schmitz and Zhang, 2011; Ji et al., 2015). Randomly fragmented DNA that was subjected to immunoprecipitation (mCIP—methyl-CpG-immunoprecipitation) coupled with tiling arrays was used for the first genome-wide analysis of DNA methylation in *A. thaliana* (Zhang et al., 2006). This method allows for an enrichment of highly methylated regions; however, it does not identify the methylation status of particular cytosines. Moreover, it was shown that mCIP is biased toward heavily methylated regions, especially those containing CHG methylation (Lister et al., 2008). In 2008, the first complete *A. thaliana* methylomes were published and served as a reference for all plant species (Cokus et al., 2008; Lister et al., 2008). Analyses were based on large-scale bisulfite sequencing (methylC-seq), which is currently the most advanced, direct and specific approach. MethylC-seq allows for the identification of the level and pattern of methylation of specific cytosines within the whole genome. Nevertheless, methylC-seq is feasible only in model species that have small and simple genomes and low repetitive element content. This technique is also costly, especially for species with genomes that are significantly larger than *Arabidopsis* or rice. Moreover, because crop cereals have large, repetitive genomes with high levels of DNA methylation in all sequence contexts (CG, CHG, and CHH), data analysis is challenging. For species with large and complex genomes (typical for many crops), indirect methods to analyze DNA methylation are commonly used due to their simplicity and cost-effectiveness. Among the currently available techniques used to determine DNA methylation modulation, Methylation Sensitive Amplification Polymorphism (MSAP) is the most widely used. MSAP is a modification of the Amplified Fragment Length Polymorphism technique (AFLP; Vos et al., 1995) and utilizes cleavage with the methylation-sensitive restriction enzymes *Hpa*II or *Msp*I, followed by adapter ligation, amplification, and further gel-based visualization (Reyna-Lopez et al., 1997; Xiong et al., 1999). The cleavage capacities of *Hpa*II and *Msp*I are strongly affected by the methylation state of the external and internal cytosine residues within the recognized 5′-CCGG-3′ sequences. Thus, the methylation state can be determined for specific bands based on the ability of each enzyme to cleave the restriction site. MSAP-based analyses can be performed for a range of species regardless of their genome size and reference genome availability. The MSAP method was established in 1997 (Reyna-Lopez et al., 1997) and has been effective in many global analyses of DNA methylation in various plant species (Xiong et al., 1999; Peraza-Echeverria et al., 2001; Chakrabarty et al., 2003; Portis et al., 2004; Filek et al., 2006; Salmon et al., 2008; Tan, 2010; Wang et al., 2011; Guzy-Wrobelska et al., 2013; Marconi et al., 2013; Tang et al., 2014). This method is still widely used in model and non-model plants (*A. thaliana*—Li et al., 2015; Xu et al., 2015; *Allium sativum*—Gimenez et al., 2016; *Brassica napus*—Gautam et al., 2016; *Gossypium hirsutum*—Wang et al., 2016; *Malus* × *Domestica*—Kumar et al., 2016; *O. sativa*—Li et al., 2017; *Vicia faba*—Abid et al., 2017). Aside from its simplicity and usefulness, MSAP only provides a general overview of the methylation state and does not provide a specific sequence context.

In the present study, we aimed to develop a simple, high-throughput, and low-cost method for the direct identification of specific genomic sequences that undergo DNA methylation in plants with large genomes. We have designed and introduced a novel technique called Methylation Sensitive Amplification Polymorphism Sequencing (MSAP-Seq) for the analysis of DNA methylation patterns in *Hordeum vulgare* (Chwialkowska et al., 2016). This method is based on the conventional MSAP analysis, which was greatly improved by replacing the conventional separation of MSAP amplicons on polyacrylamide gels with direct high-throughput sequencing using Next Generation Sequencing (NGS) and automated data analysis. MSAP-Seq allows for the global and direct identification of a large set of sequences that undergo DNA methylation changes without laborious band excisions, reamplification, and subcloning, which are required for MSAP analysis. MSAP-Seq is a simple method that allows for the parallel identification of hundreds of thousands of sites at a low cost. The complexity of the assay is reduced by subsampling only the specific sites that are cut by restrictases and amplified with selective primers. In contrast to the expensive and complex MethylC-seq analysis, our method is well-suited for analyses with large sample sets. Because MSAP-Seq relies on methylation-sensitive restriction enzymes that recognize CCGG sites, only changes within these regions are identified. Therefore, other sequence contexts, which might also undergo important changes, are not detected. Consequently, the major limitation of MSAP-Seq is that it provides only a general overview of DNA methylation modulation within selected CG sites. We have optimized the MSAP-Seq method for the popular Illumina next generation sequencing platform. To complement the MSAP-Seq methodology, we developed the MSEQER software for automated MSAP-Seq data analysis (Korotko, 2017—personal communication). Reads are mapped to the reference genome to identify specific genomic sequences and their features. Changes in DNA methylation that are identified with MSAP-Seq are characterized qualitatively (as in conventional MSAP), based on the presence or absence of each amplicon, as well as quantitatively, by deep sequencing of the MSAP-Seq amplicons, which provides the fold change values of the abundance of normalized reads. MSAP-Seq can be used by researchers that utilize the traditional MSAP analysis. Our previous experiments with MSAP as well as MSAP-Seq revealed their comparability (Chwialkowska et al., 2016) and affirmed that MSAP-Seq can be reproducibly used in studies that are designed for traditional MSAP. MSAP-Seq can be easily applied to different plant species, even those with large and complex genomes, since (1) MSAP-Seq targets gene-rich regions and the most functionally important region of the methylome, due to restriction enzymes that recognize CCGG sequences, which are more frequent in genic than other genomic regions; (2) Application of selective primers enables the adjustment of the number of generated fragments and, consequently, for the selective analysis of a smaller portion of the methylome. This feature makes the method affordable and applicable for different experimental layouts in a wide variety of species, regardless of genome size. (3) The methodology is based on the widely used (especially in crops) MSAP technique, which has been successful in various model and non-model plants. Therefore, MSAP-Seq may prove valuable in other methylome analyses, such as estimating the influence of different factors on DNA methylation or methylome diversity analyses among different genotypes or populations.

## Materials and methods

### Plant material

We collected tissues from roots and second leaves of *H. vulgare* cv. “Karat” seedlings from three time points during water-deficiency treatment under strictly controlled conditions (described in detail in Chwialkowska et al., 2016). Plants were grown in a greenhouse (20/18°C; 16/8 h photoperiod) in pots filled with a mixture of clay and sand (7:2 ratio) with known physicochemical properties. The soil moisture was measured daily in each pot using a Time-domain Reflectometer EasyTest (Institute of Agrophysics, Polish Academy of Sciences, Poland) and water was applied to maintain a 12% volumetric water content (vwc) during the control phase (time point 1), which included the first 10 days after the pre-germinated seeds were sown in pots. On day 11, the soil moisture was decreased by withholding irrigation to attain 1.5–2% vwc (drought-stressed plants, time point 2). When the soil moisture of the water-stressed plants reached 3% vwc, plants were moved to a growth chamber with a temperature regime of 25 and 20°C during the day and night, respectively. Water deficiency treatment was maintained for 10 d. Afterwards, the plants were put back into the greenhouse and normal irrigation (12% vwc) was applied for 14 d (re-watering phase, time point 3). The detached tissue samples were immediately frozen in liquid nitrogen and stored at −80°C for DNA isolation.

### DNA isolation

Genomic DNA was isolated using the micro C-TAB procedure from Doyle and Doyle (1987). The yield and purity of the DNA samples were determined using a NanoDrop ND-1000 spectrophotometer (Thermo Scientific, Waltham, USA). The integrity of the gDNA was evaluated using agarose gel electrophoresis.

### Identification of differentially methylated sites (DMS) using MSAP-Seq

Five hundred nanograms of genomic DNA was cut with 2.5 U of the frequent-cutting methylation sensitive restriction enzyme *Hpa*II (New England Biolabs, Ipswich, USA) and 2.5 U of the rare-cutting *Eco*RI (New England Biolabs, Ipswich, USA) in a 20 μL reaction with 1x NEB1 buffer (New England Biolabs, Ipswich, USA) at 37°C for 6 h. Enzymes were inactivated at 80°C for 20 min. Next, 12 μL of ligation mixture containing 60 pmol of *Hpa*II-related adapter, 6 pmol of *Eco*RI-related adapter (Table 1), 1x T4 ligase buffer (Thermo Scientific, Waltham, USA) and 1.2 U of T4 DNA ligase (Thermo Scientific, Waltham, USA) were added. This mixture was incubated at 37°C for 16 h. After that step, 5 μL of the ligation reaction was used for selective PCR amplification in a 51 μL reaction containing 75 ng of primers E-AC, 75 ng of primers H-TG (Table 1), 10 pmol of dNTPs, 5 U of Dynazyme II polymerase and 5 μL of dedicated 10x buffer (Thermo Scientific, Waltham, USA). PCR was performed under the following conditions: 30 s denaturation at 94°C, 40 s of annealing at 56°C and 50 s of extension at 72°C, for 30 cycles. Ten microliters of the amplification reaction was run on a 1.5% agarose gel. The remaining 40 μL of amplification reaction was purified using 1.8x Agencourt AMPure XP (Beckman Coulter, Brea, USA) and eluted into 50 μL 1x TE. To create tags, amplicons were fragmented with Bioruptor Plus (Diagenode, Liège, Belgium) using 10 cycles of 30 s ON followed by 30 s OFF under low power to obtain fragments with mean sizes of 300 bp. Fragmented tags were purified with 1.8x Agencourt AMPure XP (Beckman Coulter, Brea, USA) and eluted into 35 μL ddH_2_O.

**Table 1:** The sequences of the primers and adapters that were used for the MSAP-Seq analyses.

**Type**	**Name**	**Primer/oligo sequence**
Adapter *Eco*RI	EcoRI_ A1	CTCGTAGACTGCGTACC
	EcoRI_ A2	AATTGGTACGCAGTCTAC
Adapter *Hpa*II	HpaII_A1	GACGATGAGTCTAGAA
	HpaII_A2	CGTTCTAGACTCATC
PCR primer *Eco*RI-AC	E-AC	GACTGCGTACCAATTCAC
PCR primer *Hpa*II-TG	H-TG	GATGAGTCTAGAACGGTG

Sequencing libraries were subsequently prepared using the NEXTflex Rapid DNA-Seq Kit (BIOO Scientific, Austin, USA) according to the manufacturer's instructions with minor changes (option 1). Briefly, 32 μL of fragmented amplicons were added to 15 μL of end repair and adenylation buffer and 3 μL of end repair and adenylation enzyme mix. This mixture was incubated on a thermocycler under the following conditions: 22°C for 20 min, 72°C for 20 min and then 4°C on pause. Next, the barcoded adapters were ligated by adding 47.5 μL of ligase enzyme mix and 2.5 μL of undiluted DNA barcode adapter to 50 μL of end-repaired and adenylated DNA fragments. Reactions were incubated on a thermocycler for 15 min at 22°C. The ligation products were purified twice with Agencourt AMPure XP (Beckman Coulter, Brea, USA); first with 0.6x beads and an elution into 52 μL of resuspension buffer, and second using 50 μL of sample with 0.8x beads and an elution into 22 μL of resuspension buffer. Prepared fragments were subjected to PCR amplification in a total volume of 50 μL, including 5 μL of sample, 12 μL of dedicated PCR master mix and 2 μL of primer mix. PCR was performed in a thermocycler under the following conditions: initial denaturation at 98°C followed by 6 cycles of 98°C for 30 s, 65°C for 30 s and 72°C for 60 s, with a final elongation at 72°C for 4 min and then 4°C on pause. Finally, amplicons were purified with 0.8x Agencourt AMPure XP (Beckman Coulter, Brea, USA) and eluted into 21 μL of resuspension buffer. The quality of the prepared Illumina libraries was analyzed using Agilent Bioanalyzer and the Agilent High Sensitivity DNA Kit (Agilent Technologies, Santa Clara, USA), and the quantities were estimated using a Qubit Fluorometer (Thermo Fisher Scientific, Waltham, USA). For cluster generation, the libraries were diluted to 15 pM and were sequenced using the Illumina HiSeq 1500 system (Illumina, San Diego, USA) with 24 barcoded samples per lane.

### MSAP-Seq data analysis

Sequencing read processing and differential methylation analysis was performed using the automatic pipeline MSEQER (available at: http://mseqer.us.edu.pl). Reads were filtered based on the presence of the *Hpa*II-related adapter. Only reads with the *Hpa*II-related adapter were retained for further analysis. Next, reads were trimmed using BBDuk software, and those with a CGG sequence on the 3′ or 5′ in addition to a minimum length of 50 bp were retained. Reads were then mapped to the *H. vulgare* genome version ASM32608v1.31 using Bowtie2 (Langmead and Salzberg, 2012). Reads that mapped to the same CCGG location in the genome were counted and normalized using RPM (reads per million). Reads were annotated using information from the Ensembl Plants database (Yates et al., 2016) and classified into four categories: genes [classified from transcription start site (TSS) to the end, including exons and introns], putative promoters (1,000 bp upstream of the TSS), repeat regions and intergenic regions (without annotation in the genome used in the study). Then, reads were annotated functionally based on gene ontology (GO). Hierarchical clustering was performed to enable sample comparison. Differences in the levels of methylation among the samples were calculated as fold change values for normalized read counts. Statistical analysis was performed using DESeq2 software (Love et al., 2014). Data were gathered and further processed using Microsoft Excel 2016. The raw MSAP-Seq data were deposited into the SRA repository under the accession numbers PRJNA407808 and PRJNA407754, which correspond to datasets from case study 1 and 2, respectively.

### Quantitative validation using single-locus DNA methylation assay—methylation sensitive restriction enzyme qPCR (MSRE-qPCR)

Equal amounts of genomic DNA (1.25 μg) were digested with 12.5 U of *Hpa*II in 1x NEB1 buffer (New England Biolabs, Ipswich, USA) at 37°C overnight. The enzyme was inactivated at 80°C for 20 min. Mock (undigested) samples were treated simultaneously without the addition of restriction enzyme. All digests were performed in duplicate. After cleavage, samples were diluted by 5x and used as templates for qPCR amplification in a Roche LightCycler® 480 System (Roche, Basel, Switzerland) following the manufacturer's instructions with primers that flank the cut-sites (Supplementary Table S1). Amplification reactions were performed in duplicate, and their specificity was determined using a melting curve analysis. Raw data were processed using LinRegPCR (Ramakers et al., 2003). The relative methylation level was calculated using the formula FC = E^∧^ΔCp, where E is the mean amplification efficiency of a given gene and ΔCp corresponds to the difference between the Cp-values for a specific region, using the digested and undigested gDNA templates. Differences in relative methylation were analyzed using the Student's *t*-test at *P* ≤ 0.05 using STATISTICA 10 software (StatSoft).

## Results and discussion

### Technical overview of the MSAP-Seq method

**Figure 1:**
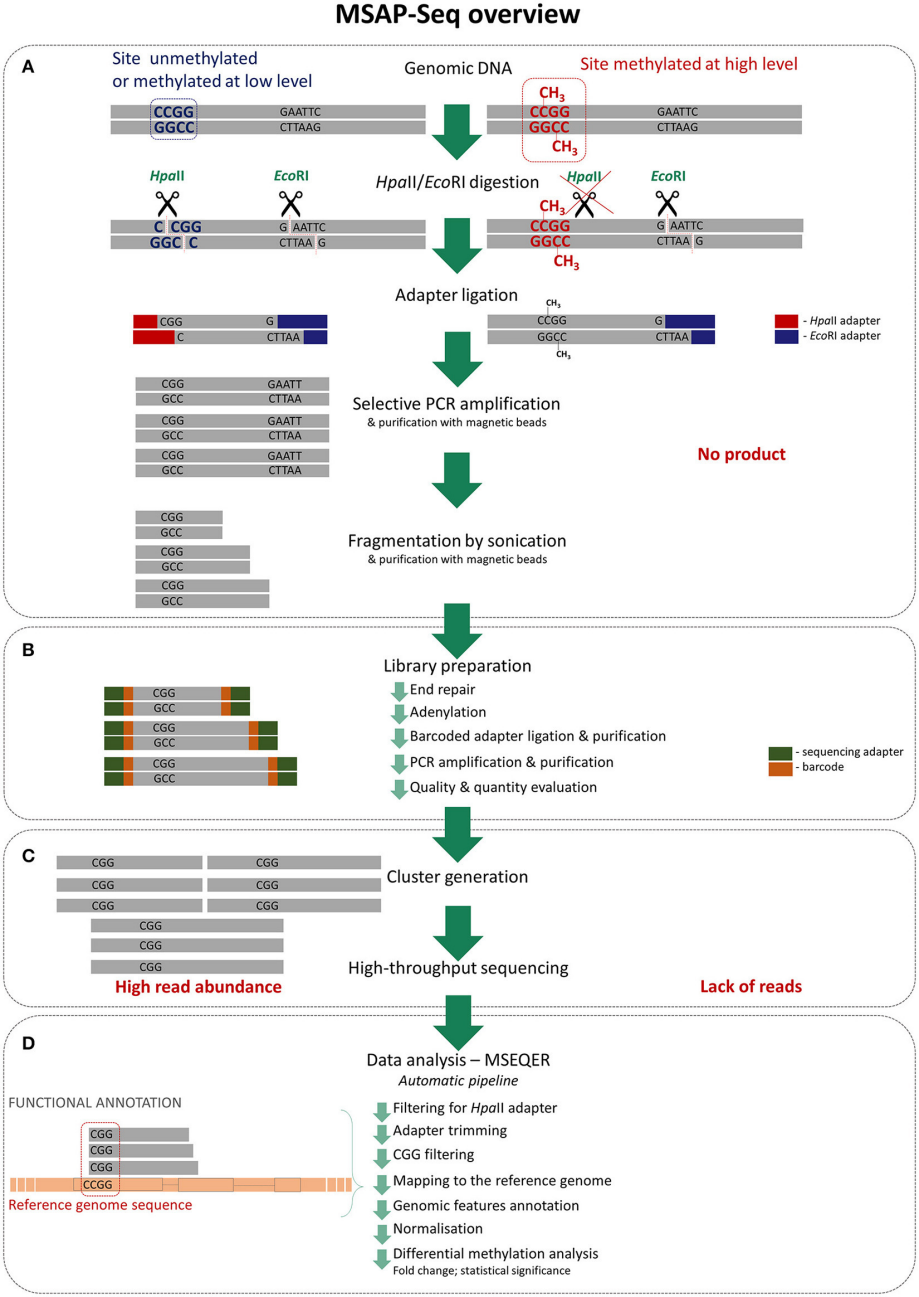
Detailed MSAP-Seq assay overview **(A)** Genomic DNA is cleaved using rare cutter (*Eco*RI) and methylation sensitive restriction enzyme (*Hpa*II); only unmethylated recognition sites are digested by *Hpa*II. Then adapters specific to sticky ends are ligated and obtained fragments are amplified in PCR using selective primers; only fragments generated from unmethylated regions are amplified as they contain ends complementary to adapters. Products are then purified and fragmented by sonication to create shorter tags. **(B)** Purified fragments are used for standard library preparation involving following steps: end repair, adenylation, barcoded adapters ligation and purification, PCR amplification and purification. Then libraries quality and quantity is estimated. **(C)** Prepared libraries are pooled and processed thru cluster generation and high-throughput sequencing. **(D)** Sequencing data are analyzed using dedicated automatic pipeline—MSEQER. Firstly, reads are filtered for presence of *Hpa*II adapter and adapters are clipped. Then only reads containing CGG tags on the ends are mapped to the reference genome and functionally annotated. Obtained counts at each of the CCGG sites are normalized and differential methylation analysis among sets of samples is performed.

MSAP is a common method used to evaluate DNA methylation in a wide range of plant species due to its simplicity, reliability and low cost. We greatly improved this method by applying the direct sequencing of amplicons using next-generation sequencing. As in typical MSAP, genomic DNA is first cut with the methylation-sensitive restriction enzyme *Hpa*II and the rare-cutter *Eco*RI, which is not sensitive to DNA methylation (Figure 1). Next, specific adaptors that are complementary to the sticky ends generated by the restrictases are ligated and amplified with partially selective primers. The number and type of selective nucleotides can be easily adjusted to the species and application. With MSAP-Seq, amplicons are sequenced using large-scale NGS, such that the number of selective primers can be greatly reduced to obtain more sequences in the final analysis. We applied two selective nucleotides, equal to 16 primer combinations, with three selective nucleotides. Thus, only one PCR amplification step is necessary in MSAP-Seq. Amplicons were then subjected to fragmentation using sonication with pre- and post-fragmentation purification, to create short tags that can be sequenced using high-throughput sequencing. Rapid DNA library sequencing was performed using the following steps: end repair, adenylation, ligation of barcoded adapters, purification, PCR amplification and purification. Libraries were later evaluated based on product quantity and size distribution. Selected libraries were then subjected to cluster generation and high-throughput sequencing using the Illumina Hi-Seq platform. Using this platform, up to 24 differentially barcoded samples can be simultaneously sequenced, allowing MSAP-Seq to be a relatively low cost method. Data are obtained easily and can be processed with the user-friendly automatic pipeline MSEQER, which is software for MSAP-Seq that was developed by our group (Korotko, 2017—personal communication). MSEQER is available as a web-based service at www.mseqer.us.edu.pl. Single-end and paired-end reads can be analyzed in single or duplicate samples. Automatic analysis allows for the filtering of *Hpa*II adapters, CGG sequence presence, adapter trimming, mapping to the reference genome, and includes genomic features and functional annotation. Read counts are then normalized and differential methylation analysis is performed among samples to obtain the FC (fold change) and *p*-values for statistical significance. The results are visualized in tables and graphs. A detailed and ready to use MSAP-Seq protocol is enclosed in Supplementary File 1. Additionally, MSAP-Seq is not restricted to species with sequenced reference genomes, as reads can be analyzed *de novo* without genome mapping for the quantitative comparison of tags among samples.

### MSAP-Seq tag sequencing using the illumina platform

To validate this method and to evaluate the biological importance of MSAP-Seq data, we performed two studies that analyzed changes in DNA methylation using MSAP-Seq: (1) an analysis of changes in the barley methylome in leaves after water-deficiency stress and (2) a comparative analysis of DNA methylation in barley leaves and roots after water deficiency stress. All analyses were performed according to our previously described methods and the detailed MSAP-Seq protocol (Supplementary File 1). Both assays were performed in barley, which is representative of large genome crops and has a complex genome that is 5.3 Gbp (Ensembl Plants; Yates et al., 2016). We also found that the barley genome was densely methylated within CCGG sites (Chwialkowska et al., 2016).

### Case study 1: analysis of changes in the barley methylome in leaves under water-deficiency stress

Our goal was to characterize the modulation of the methylome in barley leaves under conditions of water deficiency. We employed MSAP-Seq to identify changes in DNA methylation and compared three time points during drought stress: (1) control (normal watering); (2) drought (water deficiency treatment); (3) re-watering (after drought recovery with normal watering). In each time point, leaf material was harvested in triplicate, and MSAP-Seq analysis was performed as described.

**Figure 2:**
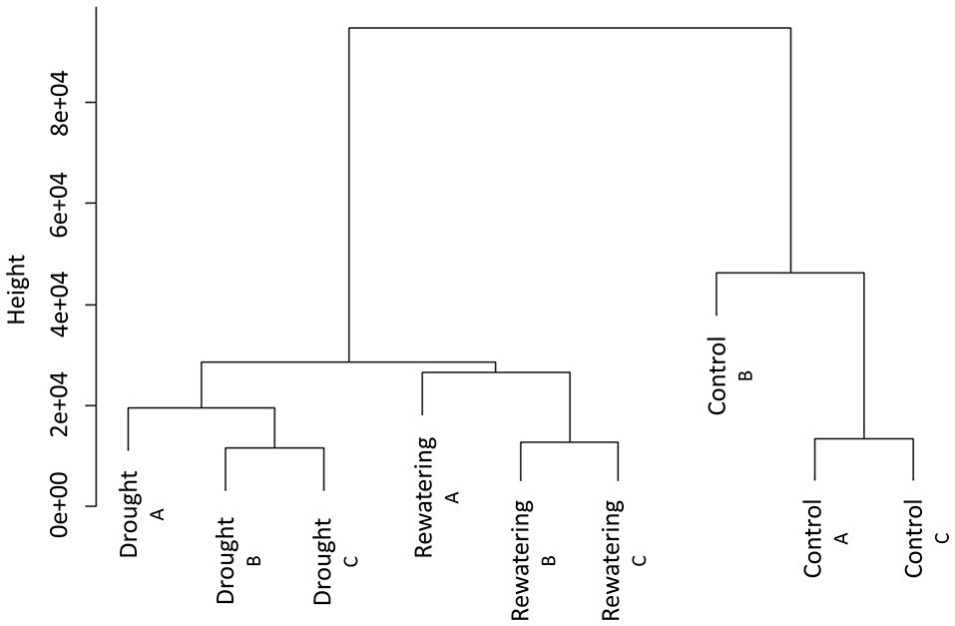
Hierarchical clustering of MSAP-Seq data of three samples (control, drought, and re-watering) performed in three independent biological replicates.

We sequenced MSAP-Seq tags with the Illumina Hi-Seq 1500 system with 24 barcoded samples per lane. We obtained 10–20 mln paired-end reads per sample (Table 2). Reads were processed with the MSEQER pipeline. Five to ten million reads were filtered based on the presence of the *Hpa*II adapter followed by a CGG sequence in addition to a read length greater or equal to 50 bp. Analyzed reads were between 50 and 91 bp with a modal value of 88 bp. Approximately 85% of reads were mapped to the barley reference genome. Not all reads could be mapped because the barley genome is incomplete and is still being assembled. On average, ~80,000 unique CCGG sites with a coverage minimum of 2 reads per sample were identified. Among them, 11,000 were gene-related sites, of which 80% were located within gene bodies and 20% within promoter regions. Clustering analysis of normalized reads counts showed that in each analyzed time point, biological replicates were similar and created one cluster (Figure 2). Separate clusters were formed for each time point. Thus, MSAP-Seq analysis was reproducible among biological replicates and allowed for the discrimination of different treatments.

**Table 2:** The statistics of MSAP-Seq reads during different data processing steps during evaluation of changes in barley methylome in leaves under water-deficiency stress.

	**Control A**	**Control B**	**Control C**	**Drought A**	**Drought B**	**Drought C**	**Re-watering A**	**Re-watering B**	**Re-watering C**	**Mean**
Initial number of reads [mln]	14.16	19.88	9.57	16.45	14.17	15.18	16.62	10.10	13.66	14.42
Number of reads after filtering^*^ [mln]	6.96	10.3	5.10	6.60	6.47	6.50	7.50	5.22	6.58	6.81
Percentage of mapped reads [%]	86.9	86.7	86.5	84.3	84.7	84.3	85.4	84.3	84.9	85.3
Number of different sites	65,980	79,929	52,164	91,374	86,706	89,271	85,101	74,211	84,738	78,830
Number of different genes (gene bodies)	7,437	8,351	6,333	9,403	9,311	9,470	9,028	8,202	8,900	8,493
Number of different genes (promoters)	1,830	2,140	1,910	2,418	2,430	2,417	2,372	2,083	2,344	2,216

* *Filtering based on HpaII-related adapter presence followed by CGG sequence and 50 bp of minimal read length*.

In total, approximately 190,000 different CCGG sites of known sequence and genomic location were identified and subjected to differential methylation analysis. This value is extremely high compared to traditional MSAP, where hundreds or thousands of amplicons are scored (Filek et al., 2006; Candaele et al., 2014; Chwialkowska et al., 2016). In MSAP, a limited quantity of differentially methylated bands are reamplified, subcloned and sequenced using the Sanger method, making this approach laborious and low-throughput. Importantly, we applied two selective nucleotides in the amplification step, equal to 16 primer combinations, compared to three selective nucleotides used in conventional MSAP. By adjusting the number of selective nucleotides in the amplification step, the number of amplicons is modified. Additionally, NGS-based sequencing with MSAP-Seq avoids the problem of homoplastic bands, which is common in gel-based MSAP studies (Vekemans et al., 2002).

**Figure 3:**
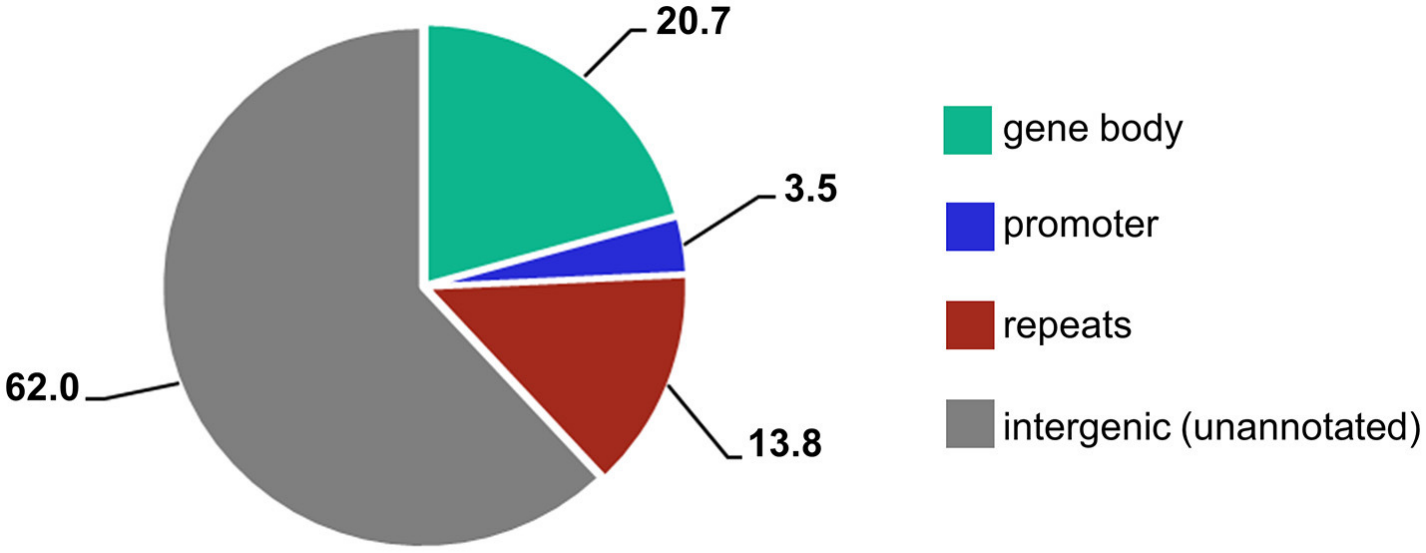
Percentages of different genic features identified within MSAP-Seq tags.

When different genomic loci were accounted for, the majority of reads (62%) mapped to unannotated intergenic regions and 14% mapped to repetitive elements (Figure 3). However, because 84%of the barley genome are mobile elements and other repeat structures (International Barley Genome Sequencing Consortium, 2012), and detailed information of repetitive element annotation is not publicly available, we annotated only those present in the Ensembl Plants database. Thus, the vast majority of the unannotated regions also presumably consists of repetitive elements. Interestingly, 25% of the sites were gene related and over 20% were located within gene bodies. Because only 2% of the barley genome contains genes, we demonstrate that the MSAP-Seq method enriches for gene-containing regions. This is because MSAP-Seq utilizes the methylation-sensitive restriction enzyme *Hpa*II, which recognizes the CCGG sequence that is frequently present in the GC-rich regions often found within genes (Glémin et al., 2014). In addition, we identified MSAP-Seq tags related to ~15,000 different genes. This is relatively high and represents ~60% of all barley genes. In conclusion, MSAP-Seq is an effective method for DNA methylation analysis for species with large genomes and low gene content.

Differentially methylated sites (DMS) were determined from normalized reads counts and the FCs among samples and the statistical significance of those changes were evaluated. The methylation-sensitive restriction enzyme *Hpa*II allowed for the distinction between two different methylation states within CCGG sequences: (1) unmethylated, which indicates that DNA is totally unmethylated or that methylation is present only on one strand (cleaved by *Hpa*II), and (2) methylated, in which the cytosine is methylated on both strands (not cleaved by *Hpa*II). The lower the read abundance is, the higher the DNA methylation level is, and conversely, the higher the read abundance is, the lower the DNA methylation level is. The normalized read counts were compared between the two datasets and changes were presented as FC-values. We identified 2,718 different CCGG sites that underwent DNA methylation changes under water-deficiency stress in barley leaves (*P* < 0.05). Fifty five percent of drought-related DMS were demethylation events and 45% were novel DNA methylations. Upon re-watering, 40% of demethylations and 50% of the new methylation events were reversible and reverted to their initial level, which was the same as the control. Within the drought-related DMS, we identified 219 new methylations and 288 demethylations that were located within genes. In both sets, 86% of the gene-related DMS were located within genes and 14% were within promoter regions. Interestingly, when the distribution of genomic features was accounted for, we found that within the drought-related DMS, genes were underrepresented and repetitive elements were overrepresented. This result suggests that changes in DNA methylation may target repetitive regions to regulate genome stability and to balance transposable element mobilization/inactivation under stress. Transposon insertions were found to be an important factor that affected adjacent gene expression under stress in maize, a plant with a highly repetitive genome (Makarevitch et al., 2015). Moreover, studies in *A. thaliana* revealed that DNA demethylases modulate the expression of stress-responsive genes by targeting changes in cytosine methylation within transposable elements (Le et al., 2014).

### Case study 2: comparative analysis of DNA methylation changes in barley leaves and roots in response to water deficiency stress

Our previous study attempted to characterize and compare the barley methylome of leaves and roots under water-deficiency treatment, as well as during the subsequent re-watering phase, with an emphasis being placed on organ-specific changes in the DNA methylation pattern (Chwialkowska et al., 2016). This experiment involved methylome modulation analysis using MSAP-Seq in the leaves and roots under the same three time points: (1) control (normal watering); (2) drought (water deficiency treatment); and (3) re-watering (after drought recovery and normal watering). Samples at each time point were pooled from three independent plants. Each time point, as well as the leaves and root samples, were pooled and analyzed separately.

We performed MSAP-Seq using the Illumina Hi-Seq 1500 system with six barcoded MSAP-Seq samples (three time points for leaves and three time points for roots). We obtained 16–77 mln paired-end reads per sample (Table 3). Reads were processed using the automatic MSEQER pipeline. Three to nineteen million reads were filtered based on the presence of the *Hpa*II-related adapter followed by a CGG sequence and a read length equal to or greater than 50 bp. Approximately 88% of filtered reads in the leaves and 49% in roots were mapped to the barley reference genome. On average, ~100,000 unique CCGG sites with a minimum coverage of 2 reads per sample were identified. Among the sites, more than 13,000 were gene-related sites, of which 80% were located within gene bodies and 20% within promoter regions.

**Table 3:** The statistics of MSAP-Seq reads during different data processing steps during comparative evaluation of DNA methylation changes in barley leaf and root in response to water deficiency stress.

	**LEAVES**				**ROOTS**			
	**Control**	**Drought**	**Re-watering**	**Mean**	**Control**	**Drought**	**Re-watering**	**Mean**
Initial number of reads [mln]	77.52	72.00	56.46	68.66	31.18	37.13	16.05	28.12
Number of reads after filtering^*^ [mln]	18.29	19.22	11.44	16.31	7.21	8.14	3.32	6.22
Percentage of mapped reads [%]	88.3	85.9	89.4	87.9	64.1	45.3	37.9	49.1
Number of different sites	117,259	122,631	95,172	111,687	107,044	111,879	57,749	92,224
Number of different genes (gene bodies)	10,961	11,248	10,336	10,848	11,819	11,905	7,728	10,484
Number of different genes (promoters)	3,067	3,247	2,805	3,039	3,107	3,050	1,785	2,647

* *Filtering based on HpaII-related adapter presence followed by CGG sequence and 50 bp of minimal read length*.

In total, ~250,000 different CCGG sites of known sequence and genomic location were identified and subjected to differential methylation analysis. When different genomic loci in the leaves and roots were accounted for, most reads (61%) mapped to unannotated intergenic regions and 13% mapped to repetitive elements. Similarly, 25% of the reads were located within genes and 21% were gene-body related, again demonstrating the enrichment for gene-containing regions. Interestingly, regarding both leaves and roots, we identified MSAP-Seq tags related to ~18,000 genes, including 75% of all barley genes.

Differentially methylated sites were determined from normalized read counts and the FCs among samples were obtained. Because samples were pooled from three plants without replicates, DMS with FCs greater than or equal to five were considered differentially methylated. MSAP-Seq analysis revealed 2,901 DMS in leaves and 6,098 in roots that exhibited changes under the water deficiency treatment. Among these sites, 496 DMS in leaves and 1,055 in roots were located within genes. In both leaves and roots, ~85% of the gene-related DMS were located within gene bodies and 15% were in promoter regions. Within the gene-related DMS in leaves, equal amounts of novel stress-induced methylation and demethylation events were observed. However, in roots, new methylation events dominated. Plant recovery after the re-watering phase led to the reversal of the major stress-induced demethylations within genes; however, this process was more efficient in leaves than in roots. In contrast, new methylation events were much more persistent in both organs. Repetitive elements preferentially underwent demethylation in leaves and novel methylation in roots. Interestingly, we found that genic regions were subjected to balanced methylome modulation rather than completely irreversible modification. This result suggests that methylation changes induced by water deficiency impacts the expression levels of stress-responsive genes. In contrast, most of the changes observed within the repetitive elements were of a stricter nature and almost all of them were irreversible methylations or completely reversible demethylations. Such tendency might allow for a reversal of most of the demethylations that could induce transposon mobilization and allow the maintenance of novel methylations that might silence them. We identified different biological processes within the subsets of the gene-related DMS in leaves compared to roots. Using Gene Ontology (GO) enrichment analysis, we determined the biological processes that were targeted by the epigenetic machinery under water-deficiency stress in both leaves and roots. For example, in the DMS of that were reversibly demethylated under conditions of drought, we observed an enrichment of metabolic processes such as xylogalacturonan and sphingolipid metabolism and raffinose biosynthesis. These processes are important in the adaptive response to water-deficiency stress (Ng et al., 2001; Liu et al., 2007; Le Gall et al., 2015). In roots, the reversibly demethylated DMS showed an enrichment of the hypersensitive response, carbohydrate metabolism, and the positive regulation of developmental processes. In conclusion, distinct biological processes were influenced by leaf- and root-specific methylation changes, which together contributed to complex stress-response networks. We offer a comprehensive catalog of the general properties of the barley leaf and root methylome, as well as its modulation under water-deficiency stress and after re-watering.

### Quantitative validation of identified DMS

In Case Study 1, a random set of 15 DMSs were identified that exhibited novel methylations in genes under water deficiency stress in barley leaves (Chwialkowska et al., 2016) and were independently validated with a single-locus DNA methylation assay using Methylation Sensitive Restriction Enzyme qPCR (MSRE-qPCR). This method is based on the cleavage of genomic DNA with the *Hpa*II enzyme followed by qPCR with primers flanking the cut-site. MSRE-qPCR was performed in two technical and three separate biological replicates that allowed for statistical analysis of the results. DNA methylation level changes exhibited similar tendencies and were comparable (Supplementary Table S2). This result demonstrates the reproducibility and reliability of detecting DMSs using MSAP-Seq.

## Conclusions

We developed and validated the MSAP-Seq method, which provides sequence-based identification of changes in DNA methylation. MSAP-Seq can easily be applied to different plant species, including those with large, complex and highly repetitive genomes. MSAP-Seq is based on conventional MSAP analysis and relies on the differential cleavage of the methylation sensitive restrictase *Hpa*II. This technique enables the fast, global, and reliable analysis of MSAP amplicons without laborious PAGE and sub-cloning assays. Thus, MSAP-Seq is as simple as the well-known and conventional MSAP but uses state-of-the-art NGS technology that enables the high-throughput, parallel and direct analysis of DNA methylation modulation in hundreds of thousands of sites. In contrast to traditional MSAP, it allows for the quantitative determination of DNA methylation changes as well as their direct localization. Additionally, the number of sequences obtained can be easily adjusted. NGS of up to 24 samples per Illumina Hi-Seq lane allows this analysis to be affordable, even for labs that conduct gel-based MSAP. One study can identify several thousand DMSs. MSAP-Seq is also well-suited for large analyses with large sets of samples. We have shown that the MSAP-Seq method enriches for gene-containing regions and that one analysis can cover 60–75% of all genes. MSAP-Seq is a method of choice for DNA methylation analysis in crop plants with large and complex genomes, due to its simplicity and low price.

## Author contributions

KC and MK: Conceived and planned the assay; KC: Carried out all the experiments and data analyses under a supervision of MK; JK: Performed sequencing on Illumina platform; UK: Prepared pipeline for bioinformatics processing; KC: Wrote the manuscript under a supervision of MK and IS. All authors read and approved the final manuscript.

### Conflict of interest statement

The authors declare that the research was conducted in the absence of any commercial or financial relationships that could be construed as a potential conflict of interest.
