# Supplementary material for: Methylation Sensitive Amplification Polymorphism Sequencing (MSAP-Seq)—A Method for High-Throughput Analysis of Differentially Methylated CCGG Sites in Plants with Large Genomes
Source: Front Plant Sci. 2017 Nov 30;8:2056. doi: 10.3389/fpls.2017.02056 (PMC5714927; doi:10.3389/fpls.2017.02056)
Supplement: Supplementary file 1 [file Table1.docx]

Supplementary Material

**Methylation Sensitive Amplification Polymorphism Sequencing (MSAP-Seq) – A Method for High-Throughput Analysis of Differentially Methylated CCGG Sites in Plants with Large Genomes**

Karolina Chwialkowska, Urszula Korotko, Joanna Kosinska, Iwona Szarejko and Miroslaw Kwasniewski*

*** Correspondence:** Corresponding Author: miroslaw.kwasniewski@umb.edu.pl

# **Supplementary table 1**

**Supplementary Table S1. The sequences of the primers that were used for the MSRE-qPCR assay**

| **No.** | **Gene ID** | **Primer Forward** | **Primer Reverse** |
| --- | --- | --- | --- |
| 1 | AJ464414 | AGGATTCCTCACGGTGTGCT | GCAATGCAATGCAAAGATCC |
| 2 | MLOC_44743 | AGCACGGCAAGGGTTACATC | ACCTCCTGATTGGCGTCTGT |
| 3 | MLOC_76473 | CGCAGATGACGGATGTAACG | CGGTGTGATGCTTGTCGATT |
| 4 | MLOC_25536 | CCCTCTCCCCTTCGTCTTCT | GCACTTGATGGGTTCACGAG |
| 5 | MLOC_70149 | AGACACGGCTGGGTAAGAGA | CCGTGAGTAAGTGCGTTGAG |
| 6 | AK252251 | GCCACTGGCATTCCACCTAT | TCTCGCAAGAGGCTATTCCA |
| 7 | MLOC_10527 | AGATTTCGGTGTCGCAGGTT | TTTGGGTCTGTTTACAAAGGAGAG |
| 8 | MLOC_14713 | CCTCGTCGACTGCCTCAAC | CAGCCATTACGAAGCAAGGA |
| 9 | MLOC_11877 | TGCAGTTCTAAAGAAGAAGAGATTCA | GCAAGCAAATTATGGAATGGA |
| 10 | MLOC_72063 | TTCATATTCAGCAGCAACAGCA | CGTTTGCTCATCATCCAATACTT |
| 11 | MLOC_36550 | GCCATGTTCTGGAAGGACAG | AGGGAGTCACAGACGGGAAC |
| 12 | MLOC_37672 | TTGAGATTCAATTCAGCTAGCAC | GGTTTCACGTTAGGGTTTAAGG |
| 13 | AK357669 | AAACGCATCGAGCAATCTGA | CTGACCGAAACCATGCTCAA |
| 14 | morex_contig_42351 | GAGCCGTCGTCGTCTTACATC | CTTGGGGTTCTCCTGCAGTC |
| 15 | morex_contig_43608 | TTATATCCTCCGCCCACGTC | CAATCCAGGCGATCTCCAAT |
