# Supplementary material for: Methylation Sensitive Amplification Polymorphism Sequencing (MSAP-Seq)—A Method for High-Throughput Analysis of Differentially Methylated CCGG Sites in Plants with Large Genomes
Source: Front Plant Sci. 2017 Nov 30;8:2056. doi: 10.3389/fpls.2017.02056 (PMC5714927; doi:10.3389/fpls.2017.02056)
Supplement: Supplementary file 2 [file Table2.DOCX]

Supplementary Material

**Methylation Sensitive Amplification Polymorphism Sequencing (MSAP-Seq) – A Method for High-Throughput Analysis of Differentially Methylated CCGG Sites in Plants with Large Genomes**

Karolina Chwialkowska, Urszula Korotko, Joanna Kosinska, Iwona Szarejko and Miroslaw Kwasniewski*

*** Correspondence:** Corresponding Author: miroslaw.kwasniewski@umb.edu.pl

# **Supplementary table 2**

**Supplementary Table S2. Quantitative validation with single-locus DNA methylation assay using Methylation Sensitive Restriction Enzyme qPCR (MSRE-qPCR)**

| **Genes** | |  | **DNA methylation under drought (FC)^#^** | | |  | **Gene function** | |
| --- | --- | --- | --- | --- | --- | --- | --- | --- |
|  |  |  |  |  |  |  |  |  |
| **No.** | **Gene ID** |  | **MSAP-seq** | **MSRE-qPCR*** | **Genic localisation** |  | **Molecular function** | **Biological process** |
| 1 | AJ464414 |  | M (8.4) | M (2.1) | Gene-body |  | Jasmonate-induced protein | Stress response |
| 2 | MLOC_44743 |  | M (4.0) | M (2.0) | Gene-body |  | NBS-LRR disease resistance protein-like | Stress response |
| 3 | MLOC_76473 |  | M (53.1) | M (5.4) | Gene-body |  | Regulator of chromosome condensation upon UV-B light | Stress response |
| 4 | MLOC_25536 |  | M (12.8) | M (2.3) | Gene-body |  | Anthocyanidin 5,3-O-glucosyltransferase | Metabolic process |
| 5 | MLOC_70149 |  | M (8.9) | M (7.4) | Promoter |  | Thebaine 6-O-demethylase | Metabolic process |
| 6 | AK252251 |  | M (4.3) | M (2.9) | Promoter |  | Cysteine proteinase | Protein modification |
| 7 | MLOC_10527 |  | M (3.6) | M (2.1) | Gene-body |  | Serine/threonine-protein kinase | Protein modification |
| 8 | MLOC_14713 |  | M (9.0) | M (3.8) | Gene-body |  | Cycle checkpoint protein RAD1 | DNA repair |
| 9 | MLOC_11877 |  | M (5.9) | M (6.3) | Gene-body |  | Chelatase subunit ChlI | Photosynthesis |
| 10 | MLOC_72063 |  | M (13.1) | M (2.3) | Promoter |  | Polyadenylate-binding protein | RNA processing |
| 11 | MLOC_36550 |  | M (6.4) | M (8.1) | Gene-body |  | Small GTPase mediated signal transduction | Signal transduction |
| 12 | MLOC_37672 |  | M (6.4) | M (5.4) | Promoter |  | 50S ribosomal protein L13 | Translation |
| 13 | AK357669 |  | M (3.8) | M (3.4) | Gene-body |  | Unknown | Other |
| 14 | morex_contig_42351 |  | M (3.2) | M (3.8) | Gene-body |  | Unknown | Other |
| 15 | morex_contig_43608 |  | M (3.9) | M (2.0) | Gene-body |  | Unknown | Other |

# - values are presented as fold change (FC) in relation to control (with FC=1); M - methylated.

* - all values are statistically significant as determined by Student's T-test (*P*≤0.05) respectively to control.
