# Supplementary material for: Methylation Sensitive Amplification Polymorphism Sequencing (MSAP-Seq)—A Method for High-Throughput Analysis of Differentially Methylated CCGG Sites in Plants with Large Genomes
Source: Front Plant Sci. 2017 Nov 30;8:2056. doi: 10.3389/fpls.2017.02056 (PMC5714927; doi:10.3389/fpls.2017.02056)
Supplement: Supplementary file 3 [file DetailedProtocol.docx]

Supplementary Material

**Methylation Sensitive Amplification Polymorphism Sequencing (MSAP-Seq) – A Method for High-Throughput Analysis of Differentially Methylated CCGG Sites in Plants with Large Genomes**

Karolina Chwialkowska, Urszula Korotko, Joanna Kosinska, Iwona Szarejko and Miroslaw Kwasniewski*

*** Correspondence:** Corresponding Author: miroslaw.kwasniewski@umb.edu.pl

# **Supplementary file 1**

# **MSAP-Seq protocol**

**Essential reagents:**

- Sterile ddH_2_O
- *Hpa*II enzyme (10U/ µL; New England Biolabs)
- *EcoR*I enzyme (20U/ µL; New England Biolabs)
- 10x buffer NEB1 (New England Biolabs)
- Mineral oil
- T4 DNA ligase (1U/ µL; Thermo Fisher Scientific) with dedicated 10x buffer
- 5 mM dNTPs
- DyNAzyme II DNA Polymerase (2U/ µL; Thermo Fisher Scientific) with dedicated 10x buffer
- Agarose gel, 0.5x TBE buffer
- Agencourt® AMPure® XP magnetic beads (Beckman Coulter) *– remember to allow Agencourt AMPure XP Beads to adjust to room temperature before use*
- Freshly prepared 80% ethanol
- 1x TE buffer
- NEXTflex™ Rapid DNA-Seq Kit (BIOO Scientific) and NEXTflex™ DNA Barcodes
- High Sensitivity DNA kit (Agilent)
- 50 pmol/µL adapter *Hpa*II
- 5 pmol/µL adapter *EcoR*I
- 50 ng/µL primer *EcoR*I-AC
- 50 ng/µL primer *Hpa*II-TG

**The sequences of the primers and adapters :**

| **Type** | **Name** | **Primer/oligo sequence** |
| --- | --- | --- |
| Adapter *Eco*RI | EcoRI_ A1 | CTCGTAGACTGCGTACC |
|  | EcoRI_ A2 | AATTGGTACGCAGTCTAC |
| Adapter *Hpa*II | HpaII_A1 | GACGATGAGTCTAGAA |
|  | HpaII_A2 | CGTTCTAGACTCATC |
| PCR primer *Eco*RI-AC | E-AC | GACTGCGTACCAATTCAC |
| PCR primer *Hpa*II-TG | H-TG | GATGAGTCTAGAACGGTG |

**Equipment:**

- Magnetic stand for 1.5 mL tubes
- Vortex
- Thermocycler
- Sonicator – for eg. Bioruptor® Plus (Diagenode)
- Agilent Bioanalyzer or equipment for gel electrophoresis

**PROTOCOL**

1. **Restriction enzymes digestion**
   1. Prepare dilutions of your DNA samples in a minimum 10 µL volume and concentration of 50 ng/µL in ddH_2_O.
   2. Combine in a PCR tube following reagents (volumes per one sample):

7.625 µL ddH_2_O

10 µL 50 ng/µL DNA sample

2 µL 10x buffer NEB1 (New England Biolabs)

0.25 µL *Hpa*II enzyme (10U/ µL; New England Biolabs)

0.125 µL *EcoR*I enzyme (20U/ µL; New England Biolabs)

20 µL TOTAL

- 1. Incubate at 37^o^C for 6 h in a thermocycler or water bath; remember to add a drop of mineral oil if you do not use a thermocycler with heated lid.
  2. Inactivate enzymes by incubation at 80^o^C for 20 min in a thermocycler.

1. **Adapter ligation**
   1. Prepare ligation mix on ice in a separate tube (volumes per one sample):

7.2 µL ddH_2_O

1.2 µL 50 pmol/µL adapter *Hpa*II

1.2 µL 5 pmol/µL adapter *EcoR*I

1.2 µL 10x buffer for T4 DNA ligase (Thermo Fisher Scientific)

1.2 µL T4 DNA ligase (1U/ µL; Thermo Fisher Scientific)

12 µL TOTAL

- 1. Add 12 µL of the ligation mix to the post-restriction sample, mix, quick-spin (if you applied mineral oil remember to add it to the solution under the oil).
  2. Incubate at 37^o^C for 16 h in a thermocycler or water bath.

1. **PCR amplification**
   1. Prepare PCR mix on ice in a separate tube (volumes per one sample)

33.5 µL ddH_2_O

5 µL Sample after the adapter ligation step

1.5 µL 50 ng/µL primer *EcoR*I-AC

1.5 µL 50 ng/µL primer *Hpa*II-TG

1.2 µL 5 mM dNTPs

1.2 µL 10x buffer for DyNAzyme II DNA Polymerase

(Thermo Fisher Scientific)

2.5 µL DyNAzyme II DNA Polymerase (2U/ µL;

Thermo Fisher Scientific)

51 µL TOTAL

- 1. Perform PCR under the following programme:

94^o^C - 30 s

x 30 cycles

56^o^C - 40 s

72^o^C - 50 s

4 ^o^C - pause

- 1. You can examine 10 µL of sample after amplification on a 1.5% agarose gel (expect smear of bands in a range of 200-800 pz).
  2. At this step you can also pool your samples if you do not want to prepare separate biological replicates but a pools of them. Simply mix the same volumes of PCR products from each of the biological replicates and use 40 µL of such pool in the next step.

1. **Post-PCR purification with magnetic beads**
   1. Add 40 µL of sample after amplification to the 72 µL of resuspended Agencourt® AMPure® XP magnetic beads (Beckman Coulter) in a 1.5 mL tube.
   2. Mix thoroughly by pipetting for 10 times and incubate for 5 minutes at room temperature.
   3. Place the mixture onto a magnetic stand for 2 min or until the beads settle and supernatant is clear.
   4. Aspirate the cleared solution from the tube and discard.
   5. With a tube on a magnetic stand add 200 μL of 80% ethanol to each sample and incubate for 30 seconds at room temperature (do not disturb the beads). Carefully remove ethanol by pipette and discard.
   6. Repeat previous step for a total of 2 washes. Ensure all ethanol has been removed.
   7. Remove the tube from the magnetic stand and let dry at room temperature for 5 minutes or until bead pellet is visibly dry. Do not over-dry the beads (they cannot crack).
   8. Resuspend dried beads with 53 μL of 1x TE buffer.
   9. Mix thoroughly by pipetting for 10 times and incubate for 5 min at room temperature.
   10. Place the sample onto a magnetic stand for 2 min or until the beads settle and supernatant is clear.
   11. Transfer 50 μL of sample into a new tube.
   12. Cool samples in a fridge and keep on ice until fragmentation step. You can also freeze them at -20 ^o^C and thaw on ice before sonication.
2. **Fragmentation with sonication**
   1. Perform fragmentation by sonication to obtain fragments in a range of 300 bp. If using Bioruptor® Plus (Diagenode) perform fragmentation of 50 μL of sample in 1x TE under the following conditions:

LOW power

30 s ON

x 10 cycles

30 s OFF

1. **Post-fragmentation purification with magnetic beads**
   1. Add 50 µL of sample after amplification to the 90 µL of resuspended Agencourt® AMPure® XP magnetic beads (Beckman Coulter) in a 1.5 mL tube
   2. Mix thoroughly by pipetting for 10 times and incubate for 5 minutes at room temperature.
   3. Place the mixture onto an magnetic stand for 2 min or until the beads settle and supernatant is clear.
   4. Aspirate the cleared solution from the tube and discard.
   5. With a tube on a magnetic stand add 200 μL of 80% ethanol to each sample and incubate for 30 seconds at room temperature (do not disturb the beads). Carefully remove ethanol by pipette and discard.
   6. Repeat previous step for a total of 2 washes. Ensure all ethanol has been removed.
   7. Remove the tube from the magnetic stand and let dry at room temperature for 5 minutes or until bead pellet is visibly dry. Do not over-dry the beads (they cannot crack).
   8. Resuspend dried beads with 37 μL of ddH_2_O.
   9. Mix thoroughly by pipetting for 10 times and incubate for 5 min at room temperature.
   10. Place the sample onto an magnetic stand for 2 min or until the beads settle and supernatant is clear.
   11. Transfer 35 μL of sample into a new tube.
   12. Evaluate amplicon concentration so that you have around 1 μg or more of DNA fragments in 32 μL volume (maximum volume to be processed during library preparation).
2. **Library preparation**

*Protocol with the application of the NEXTflex™ Rapid DNA-Seq Kit (BIOO Scientific) compatible with Illumina sequencing systems*

1. **End repair & adenylation**
   1. Combine the following reagents on ice in a PCR tube (volumes per one sample)

32 µL Purified sample suspended in H_2_O

15 µL NEXTflex™ End-Repair & Adenylation Buffer Mix

3 µL NEXTflex™ End-Repair & Adenylation Enzyme Mix

50 µL TOTAL

- 1. Incubate on a thermocycler using the following program:

22^o^C - 20 min

72^o^C - 20 min

4^o^C - pause

1. **Adapter ligation**
   1. Thaw NEXTflex™ Ligase Enzyme Mix to room temperature then vortex for 5-10 seconds. Do not spin down tube, as this may cause components of the mix to separate and affect performance.
   2. Prepare ligation mix on ice (volumes per one sample)

*At this step you can barcode each of the samples separately; up to 24 samples can be run on one Illumina sequencing lane, so you can use 24 different barcodes.*

50 µL DNA sample after end repair and adenylation

47.5 µL NEXTflex™ Ligase Enzyme Mix

2.5 µL appropriate NEXTflex™ Barcode (from 1 to 24)

100 µL TOTAL

- 1. Thoroughly mix the reaction by pipetting up and down 15 times and visually inspect tubes to ensure proper homogenization as the ligase enzyme mix is very viscous.
  2. Incubate at 22^o^C for 15 min in a thermocycler.
  3. Mix in a 1.5 mL tube 100 µL of sample after adapter ligation and 60 μL of AMPure XP Beads to each sample. Mix thoroughly until homogenized.
  4. Incubate sample at room temperature for 5 minutes
  5. Place the tube on the magnetic stand at room temperature for 5 minutes or until the beads settle and supernatant is clear.
  6. Aspirate the cleared solution from the tube and discard.
  7. With a tube on a magnetic stand add 200 μL of 80% ethanol to each sample and incubate for 30 seconds at room temperature (do not disturb the beads). Carefully remove ethanol by pipette and discard.
  8. Repeat previous step for a total of 2 washes. Ensure all ethanol has been removed.
  9. Remove the tube from the magnetic stand and let dry at room temperature for 5 minutes or until bead pellet is visibly dry. Do not over-dry the beads (they cannot crack).
  10. Resuspend dried beads with 52μL of Resuspension Buffer. Mix thoroughly by pipetting until homogenized.
  11. Incubate for 5 min at room temperature.
  12. Place the sample onto an magnetic stand for 5 min or until the beads settle and supernatant is clear.
  13. Do not discard the sample in this step. Transfer 50 μL of clear sample to a new tube..
  14. Add 40 μL of AMPure XP Beads to each sample. Mix thoroughly until homogenized.
  15. Incubate sample at room temperature for 5 minutes or until the beads settle and supernatant is clear.
  16. Aspirate the cleared solution from the tube and discard.
  17. With a tube on a magnetic stand add 200 μL of 80% ethanol to each sample and incubate for 30 seconds at room temperature (do not disturb the beads). Carefully remove ethanol by pipette and discard.
  18. Repeat previous step for a total of 2 washes. Ensure all ethanol has been removed.
  19. Remove the tube from the magnetic stand and let dry at room temperature for 5 minutes or until bead pellet is visibly dry. Do not over-dry the beads (they cannot crack).
  20. Resuspend dried beads with 22μL of Resuspension Buffer. Mix thoroughly until homogenized.
  21. Incubate resuspended beads at room temperature for 5 minutes.
  22. Place the tube on the magnetic stand at room temperature for 5 minutes.
  23. Transfer 20 μL of clear sample to a new tube.
  24. You can pause at this step with samples stored at -20 ^o^C. To restart thaw frozen samples on ice.

1. **PCR amplification**
   1. Combine the following reagents on ice in the PCR plate (volumes per one sample):

5 µL Purified DNA sample after adapter ligation

31 µL Nuclease-free water

12 µL NEXTflex™ PCR Master Mix

2 µL NEXTflex™ Primer Mix

50 µL TOTAL

- 1. Thoroughly mix the reaction by pipetting and place in thermocycler under the following PCR programme:

98^o^C - 2 min

98^o^C - 30 s

x 6 cycles

65^o^C - 30 s

72^o^C - 60 s

4 ^o^C - pause

- 1. Mix in a 1.5 mL tube 50 µL of sample after adapter ligation and 40 μL of AMPure XP Beads to each sample. Mix thoroughly until homogenized.
  2. Incubate sample at room temperature for 5 minutes.
  3. Place the tube on the magnetic stand at room temperature for 5 minutes or until the beads settle and supernatant is clear.
  4. Aspirate the cleared solution from the tube and discard taking care not to disturb beads. Some liquid may remain in wells.
  5. With a tube on a magnetic stand add 200 μL of 80% ethanol to each sample and incubate for 30 seconds at room temperature (do not disturb the beads). Carefully remove ethanol by pipette and discard.
  6. Repeat previous step for a total of 2 washes. Ensure all ethanol has been removed.
  7. Remove the tube from the magnetic stand and let dry at room temperature for 5 minutes or until bead pellet is visibly dry. Do not over-dry the beads (they cannot crack).
  8. Resuspend dried beads with 21 μL of Resuspension Buffer. Mix thoroughly until homogenized.
  9. Incubate resuspended beads at room temperature for 5 minutes.
  10. Place the tube on the magnetic stand at room temperature for 5 minutes.
  11. Transfer 20 μL of clear sample to a new well.
  12. Examine your library distribution by gel electrophoresis or Agilent Bioanalyzer.
  13. Quantify DNA library templates with Qubit (Thermo Fisher Scientific) or using qPCR, which recommended for optimal cluster density. This can be performed using any qPCR quantification kit with the NEXTflex™ Primer Mix.
  14. The library is now ready for cluster generation with the standard Illumina protocol. Proceed to cluster generation or store at -20°C.
  15. For cluster generation the libraries should be diluted to the 15 pM and then sequenced in the Illumina system (for eg. Illumina HiSeq 1500) with a density of one samples per 1/24 lane (up to 24 samples at one lane).


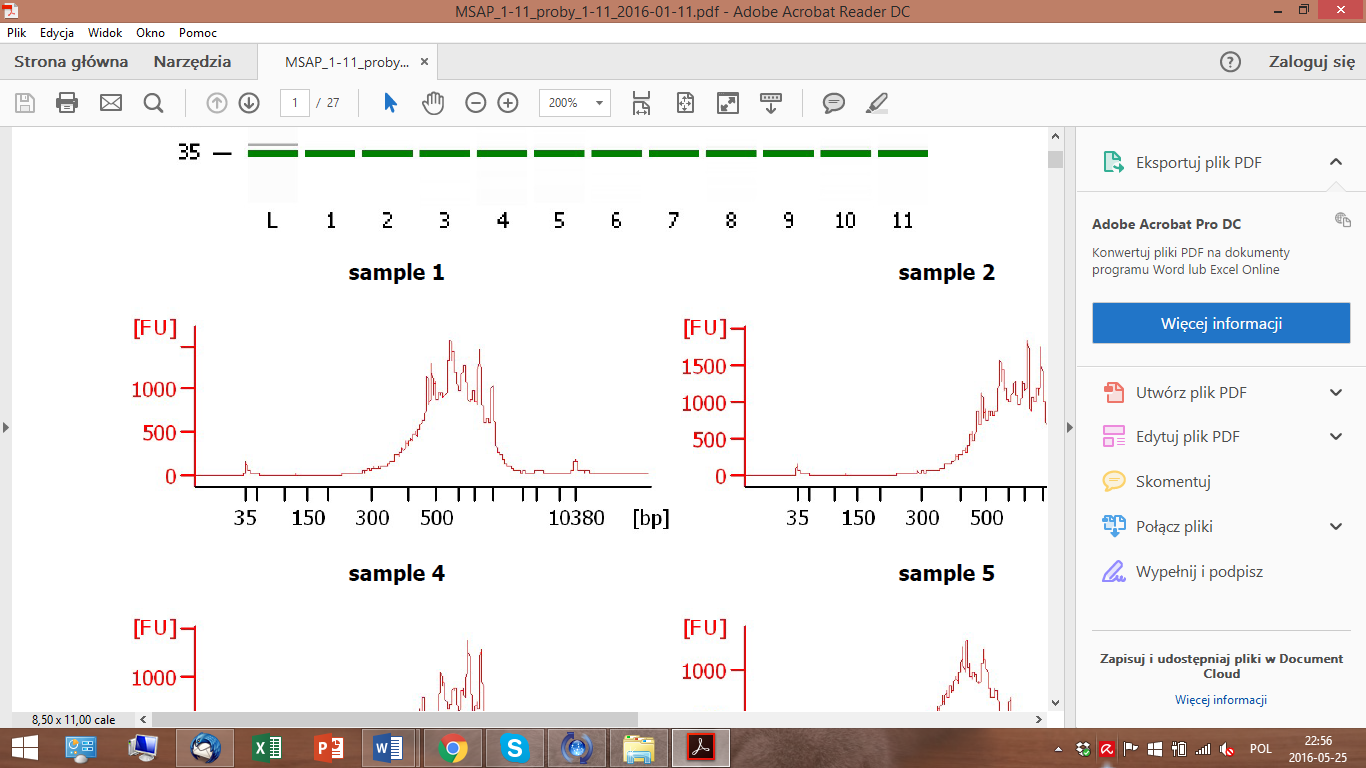

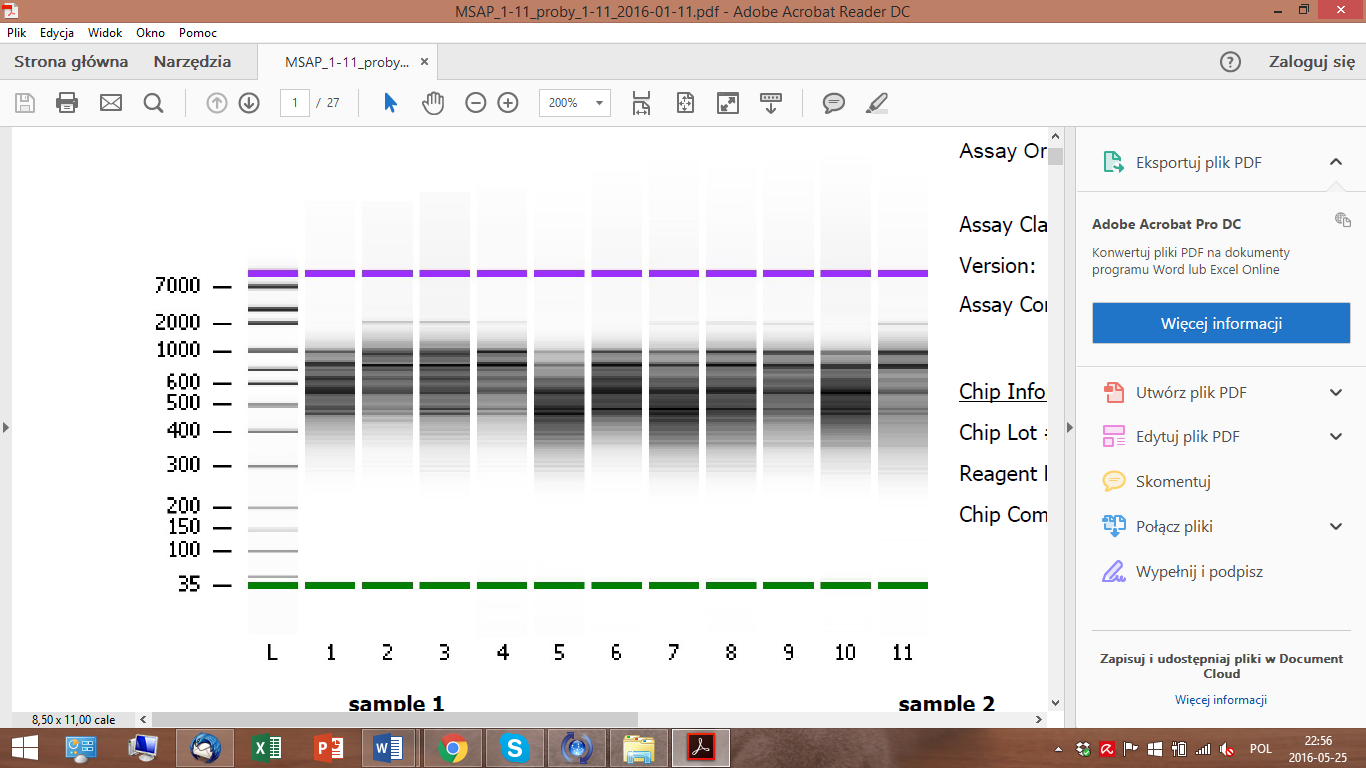


**Fig. MSAP-Seq library validation with Agilent Bioanalyzer and the High Sensitivity DNA kit (Agilent)**

1. **Data analysis**
   1. After sequencing proceed to data analysis with MSEQER software available at http://mseqer.us.edu.pl/
   2. After files uploading continue to MSAP-Seq analysis:
      1. Select appropriate reference genome
      2. If You were using paired-end reads – match pairs of files
      3. Mark biological replicates
      4. Provide sequences of adapters to be trimmed. If You are using adapters provided in the protocol paste to the left box: GATGAGTCTAGAA (Adapter *Hpa*II); and to the right box: GACTGCGTACCAATTCAC (Adapter *Eco*RI)
      5. Run the analysis
